# Technical Framework for Enabling High Quality Measurements in New Approach Methodologies (NAMs)

**DOI:** 10.14573/altex.2205081

**Published:** 2022-07-15

**Authors:** Elijah J. Petersen, John T. Elliott, John Gordon, Nicole C. Kleinstreuer, Emily Reinke, Matthias Roesslein, Blaza Toman

**Affiliations:** 1 National Institute of Standards and Technology (NIST), Gaithersburg, MD, USA; 2 US Consumer Product Safety Commission, Rockville, MD, USA; 3 National Institute of Environmental Health Sciences, National Toxicology Program Interagency Center for the Evaluation of Alternative Toxicological Methods, Research Triangle Park, NC, USA; 4 U.S. Army Public Health Center, Aberdeen Proving Ground, MD, USA; 5 Empa, Swiss Federal Laboratories for Material Testing and Research, Particles-Biology Interactions Laboratory, St. Gallen, Switzerland

## Abstract

New approach methodologies (NAMs) are *in vitro*, *in chemico*, and *in silico* or computational approaches that can potentially be used to reduce animal testing. For NAMs that require laboratory experiments, it is critical that they provide consistent and reliable results. While guidance has been provided on improving the reproducibility of NAMs that require laboratory experiments, there is not yet an overarching technical framework that details how to add measurement quality features into a protocol. In this manuscript, we discuss such a framework and provide a step-by-step process describing how to refine a protocol using basic quality tools. The steps in this framework include 1) conceptual analysis of sources of technical variability in the assay, 2) within-laboratory evaluation of assay performance, 3) statistical data analysis, and 4) determination of method transferability (if needed). While each of these steps has discrete components, they are all inter-related, and insights from any step can influence the others. Following the steps in this framework can help reveal the advantages and limitations of different choices during the design of an assay such as which in-process control measurements to include and how many replicates to use for each control measurement and for each test substance. Overall, the use of this technical framework can support optimizing NAM reproducibility, thereby supporting meeting research and regulatory needs.

## 1 Introduction

Numerous governmental policies and directives in different countries are pushing for reduced animal testing and better prediction of human health risks (EPA, 2019; United States, 2016; NCad, 2016; EU, 2010; CPSC, 2022). To address this need, *in vitro*, *in chemico*, and *in silico* (computational) new approach methodologies (NAMs) are being developed that could be used to supplement or replace *in vivo* testing. These NAMs cover different routes of potential exposure (e.g., inhalation (Lacroix et al., 2018; Zavala et al., 2020), oral (Tanneberger et al., 2010, 2013; Petersen 2022a), and dermal (Sullivan et al., 2017; Urbisch et al., 2015)) and provide information on safety and efficacy of different products and compounds. In addition to helping to reduce animal testing, NAMs have several potential advantages over *in vivo* testing such as lower costs, the ability to test specific biological mechanisms, improved understanding of assay variability, and greater similarity to human biology. NAMs are increasingly being considered for use by different agencies in the U.S. with new policies recently published (e.g., CPSC, 2022) and under development (EPA, 2019) and a new strategic roadmap published by the Interagency Coordinating Committee on the Validation of Alternative Methods (ICCVAM) (ICCVAM, 2018). Potential contexts of use for NAMs are diverse and range from screening and prioritization of large numbers of chemicals to hazard assessments for specific toxicity endpoints to providing data that can be used within quantitative risk assessments (Parish et al., 2020).

NAMs that rely upon laboratory experiments need to yield comparable results within, and potentially also among, laboratories across time to provide confidence in the long-term use of data from different experiments. Whether transferability among laboratories is required to be demonstrated prior to regulatory usage depends upon the context of use and regulatory requirements of the agencies involved (Parish et al., 2020). To achieve this aim, it is critical to add quality features that can be used to quantify, monitor, and control key sources of variability to NAMs. Guidance on methodology standards for NAMs for research and regulatory testing needs is available, such as the Organization for Economic Cooperation and Development (OECD) Good In Vitro Methods Practices (GIVIMP) guidance document (OECD, 2018), the guidance document on good cell and tissue culture practice (GCCP) 2.0 (Pamies et al., 2022), and the U.S. National Center for Advancing Translational Science (NCATS) Assay Guidance Manual (AGM) for high throughput assays (Coussens et al., 2018). While these documents list a range of important topics to be considered during NAM development, it is challenging to cover all potentially relevant considerations since these assays span a broad range of test systems (*in chemico* and *in vitro*), biological test articles (e.g., adherent cells and 3D constructs), exposure methods (e.g., aerosol exposure at an air-liquid interface), and types of substances tested (e.g., gases, medical devices, and dissolved substances). Strategies to direct the process of adding quality features to a NAM could help systematically organize the different topics listed in these guidance documents, provide a clear set of steps to follow, and describe how and when to use relevant basic quality tools.

**Fig. 1:**
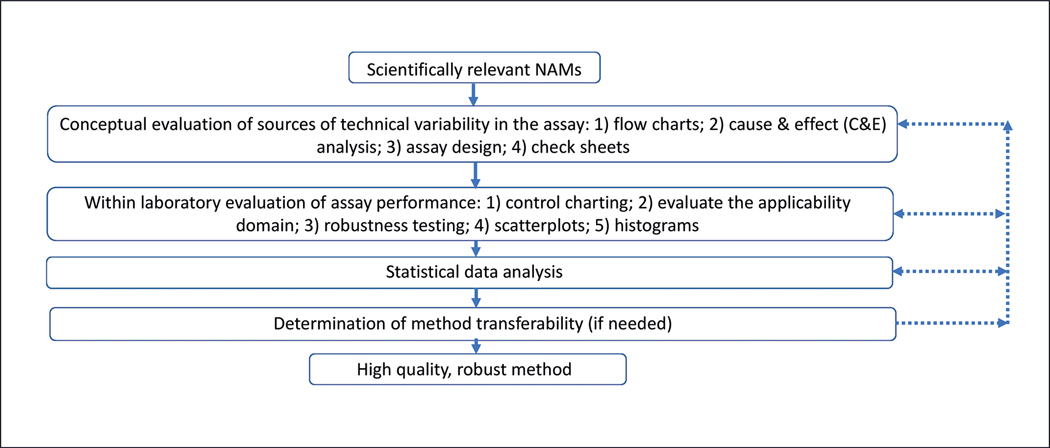
Framework for developing robust NAMs Solid lines indicate steps that should be taken in the suggested order. Dotted lines indicate a direction that can be taken, if necessary, to reevaluate any of the previous steps. For example, results from an interlaboratory evaluation could lead either to a finished method or a return to a previous step for revisions and potentially additional experiments (e.g., more experiments for the within-laboratory evaluation step). If results from a latter step require a revision to an earlier step, work may not be needed at other earlier steps. For example, results from an interlaboratory evaluation may indicate the need for more robustness testing, but revisions to the statistical data analysis and reporting and the conceptual evaluation may not be needed.

In this paper, we describe a framework for adding measurement quality features to *in vitro* and *in chemico* NAMs. Quality considerations for computation (or *in silico*) NAMs are detailed in OECD GD 69 and the associated proposed QSAR Model Reporting Format (OECD, 2014) and are not covered here. This paper focuses on understanding and minimizing technical variability (i.e., the variability when attempting to perform the same assay under the same conditions) but not biological variability (i.e., variability that stems from using primary cells from multiple donors in experiments designed to understand the range of responses within a population) (Parish et al., 2020). However, having technical variability under control can improve estimates of biological variability. Quality can be defined in different ways and is described in many papers and guidance documents. In this paper, quality refers solely to measurement quality. This framework is designed for NAMs that already have a clear context of use (e.g., the potential to fulfill a particular regulatory testing need) and biological/physiological relevance (e.g., covering a key event in an adverse outcome pathway). This framework details the specific inter-related steps that can be used to technically assess a NAM: conceptual evaluation of the assay, within-laboratory evaluation, statistical data analysis and reporting, and if needed, interlaboratory evaluation (Fig. 1). Following these steps helps ensure that the key sources of variability in the assay have been thoroughly evaluated and then mitigated or controlled to provide system performance measurements that support high confidence in the assay results. Quantifying the variability of different components of the assay (e.g., variability of pipetting cells) through in-process control measurements that are evaluated each time the assay is performed will reveal their relative contribution to the overall assay variability. This process will support making evidence-based choices in the assay design through robustness testing and statistical analysis. There may still be tradeoffs among choices when developing a protocol. For example, including additional in-process control measurements may increase the cost to perform the assay, and testing another in-process control measurement may decrease the number of test substances that can be tested in a plate. Nevertheless, this evaluation will help reveal the advantages and disadvantages of different options. Statistical characterization of the assay can yield probabilistic information based on threshold criteria in selecting a binary choice (e.g., whether the test compound has the evaluated biological effect). This strategy uses information about the assay variability each time it is performed to define test results and the probability metric supporting a decision. Within the steps in this framework, basic process quality tools originally described by Ishikawa (1985) (cause-and-effect analysis, flowcharts, check sheets, control charts, histograms, and scatterplots) are applied within the NAM development process. The seventh basic quality tool described by Ishikawa, the Pareto plot, can be used to assess the most frequent causes of assay failure, but is not discussed further here since assay developers will probably have not performed the assay often enough to have sufficient statistical power to analyze the failures. Applying these tools to the measurement process of NAMs can result in the addition of intermediate metrics, control experiments, and specifications that indicate the measurement system is performing as expected and that sources of variance are under control.

This framework is a response to prevailing challenges to ensure confidence in complex assays and studies that support NAM development. Application of the framework can increase confidence in NAM test results and address issues such as reproducibility and quality needs for consistent long-term use of the assay for regulatory decision-making processes. Increasing the technical quality of a NAM decreases the likelihood of false positive and false negative calls (see Table 1 for examples of artifacts and biases with NAMs revealed by quality control measurements). Moreover, substantial efforts to fully understand the sources of variability in test methods greatly facilitate documentary standards development.

## 2 Conceptual evaluation of sources of technical variability in the assay

The first step in ensuring quality of a NAM is to use conceptual tools. These tools do not require performing experiments and can therefore be applied more quickly. The results from the use of the conceptual evaluation can help guide the subsequent intra-laboratory evaluation of the NAM and raise confidence that the key sources of variability in the NAM are investigated and that the control measurements provide coverage of the various protocol steps.

### Flow chart: illustrate protocol steps

Flow charts diagram every step in a protocol. They can be used to ensure that all steps are performing as expected by covering and tracking each step, if feasible, through control measurements. Control measurements may cover multiple steps in a protocol, and different control measurements may cover the same step. Analyzing the information on what steps a control measurement does and does not cover may help to identify what parts of an assay are not performing as expected if some control measurements are within specifications while others are not.

**Fig. 2:**
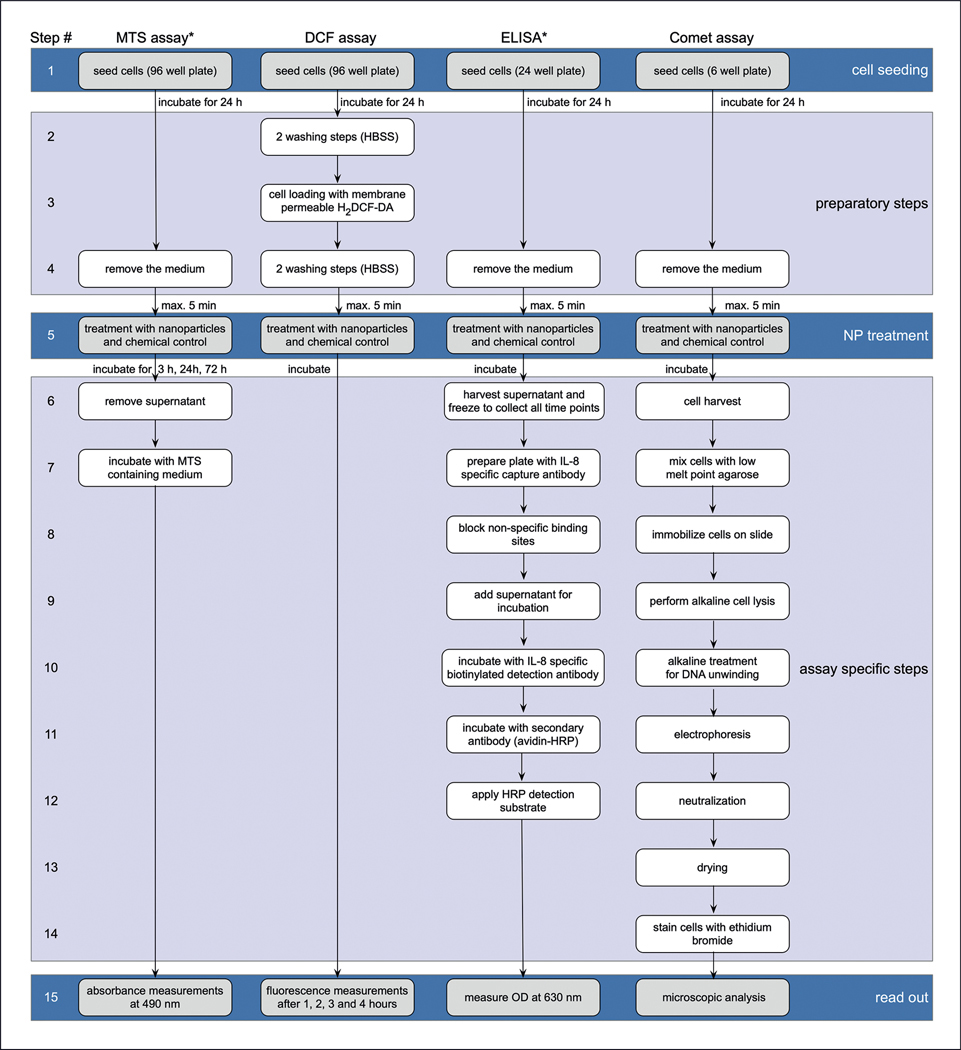
Example of flow charts illustrating steps for MTS, DCF, ELISA, and Comet assays Asterisks indicate that this method has multiple washing steps that are not elaborated for brevity. Nevertheless, these washing steps can be a source of operator mistakes. Modified and reprinted with permission from Petersen et al. (2020).

Flow charts also facilitate comparing the steps and related sources of variability among assays. Figure 2 shows flow charts comparing the steps in four *in vitro* nanobioassays: the 2’,7’-dichlorofluorescein (DCF) assay, an enzyme-linked immunosorbent assay (ELISA) for measuring interleukin-8, the 3-(4,5-dimethylthiazol-2-yl)-5-(3-carboxymethoxyphenyl)-2-(4-sulfophenyl)-2H-tetrazolium (MTS) cell viability assay, and the Comet assay (Petersen et al., 2020). It is clear that all of the assays have some steps in common (e.g., the cell seeding step), while other steps are shared by fewer assays such as the plate reader analysis (last step) by the MTS, DCF, and ELISA method. If there is an improvement to reduce the variability in a step that is shared among assays (e.g., the cell seeding step), this could help reduce the variability in all the assays. For example, if several assays require cell scraping off the bottom of the culture plate prior to cell seeding (a practice that is challenging to make more reproducible), a method that can better individually disperse the cells prior to cell seeding without scraping could be helpful.

### Cause-and-effect analysis: diagram known sources of variability

**Fig. 3:**
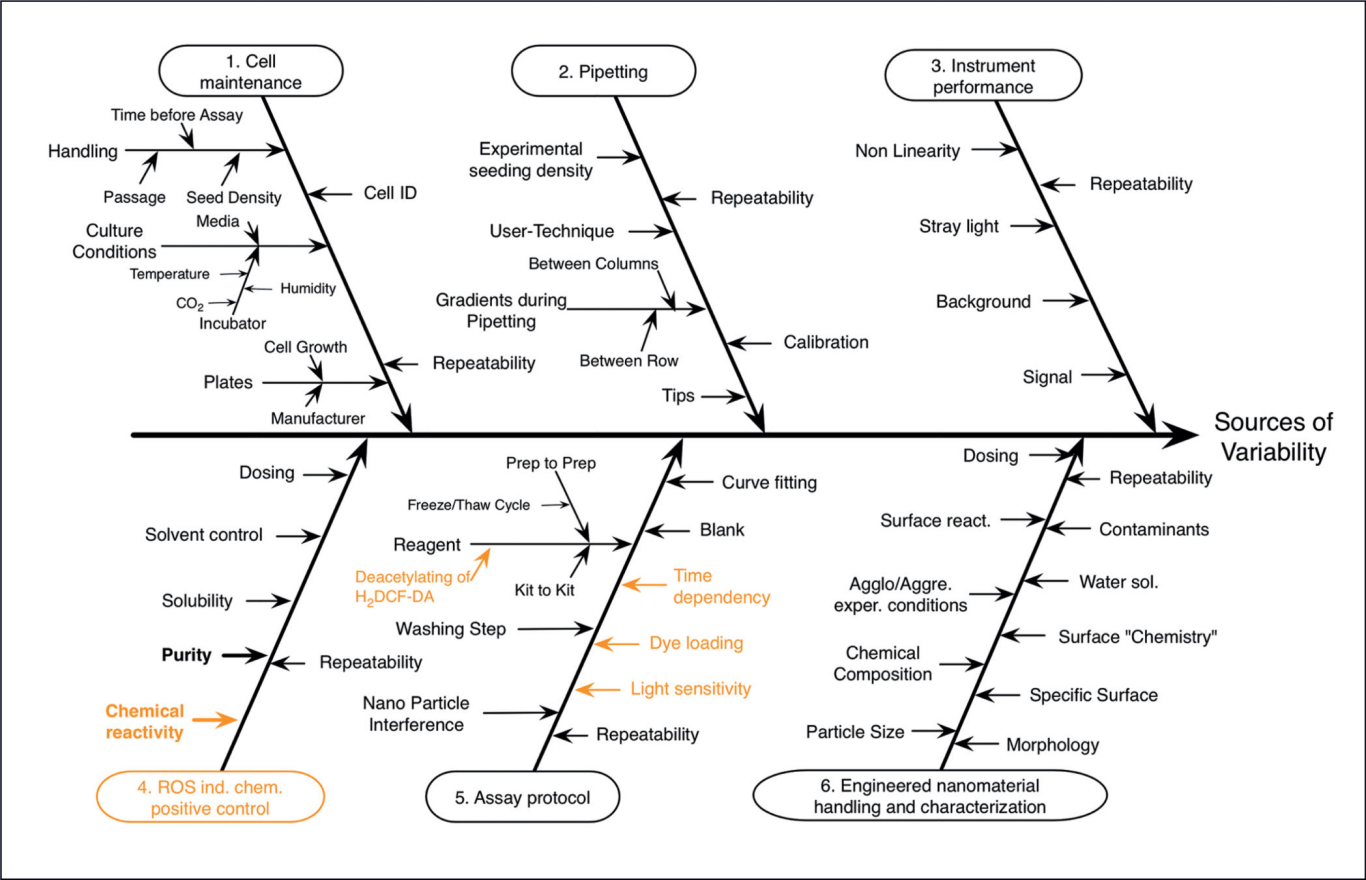
Example of a C&E diagram using the DCF assay Parts of the diagram in orange font indicate differences from the C&E diagram for the MTS assay. Reprinted with permission from Petersen et al. (2020).

Cause-and-effect (C&E) analysis is an approach that diagrams the possible key sources of variability in a method using C&E diagrams (Hanna et al., 2016; Leibrock et al., 2020; Petersen et al., 2020, 2021b; Rösslein et al., 2015). Having a visual representation of these possible sources of variability can support discussions among scientists and provide insights into the assay. Similar to the flow charts, making a C&E diagram for a new method can be done efficiently by reusing components of previous C&E diagrams where relevant as is illustrated for the DCF assay in comparison to the MTS assay (Fig. 3) (Petersen et al., 2020). This stems from the multiple overlapping steps in the flow charts for the DCF and MTS assays.

When branches of the C&E diagrams are shared among assays (e.g., the same analytical instrument used), the related sources of variability will also likely be similar, enabling sharing of variability reduction strategies (Petersen et al., 2020). For example, the use of a “bubble control” to evaluate if the presence of undetected bubbles in a well of a 96-well plate could bias the plate reader results was suggested during the standardization of the MTS assay for cytotoxicity testing of engineered nanomaterials (ISO, 2018). This control is performed by taking an absorbance measurement of each well at a wavelength outside of the absorption spectrum of the probe molecule and evaluating if this value is greater than the historical range. This bubble control has been incorporated into unrelated assays that share a common branch in the C&E diagram: each assay uses a plate reader.

In addition, C&E analysis may help reveal limitations of a method. For example, this analysis may reveal reagents that are likely to have substantial variability among batches or manufacturers or are unstable. The DCF assay tests the ability of a compound to produce reactive oxygen species (ROS) (Fig. 3), so positive control materials for this assay need to generate ROS. However, such compounds are unstable, challenging to store and quantify, and the concentration may change during the course of the assay preparation (Roesslein et al., 2013). Further, C&E analysis for the instrument branch may show when an instrument is challenging to calibrate or ensure measurement transferability, which may limit its potential for widespread use.

Ideally, experiments should be performed to understand the sources of uncertainty in each branch and subbranch of the C&E diagram. However, proprietary elements, such as software used to analyze the assay output, may hinder fully understanding the contributors to assay variability if these components cannot be thoroughly vetted and understood. Understanding the variability of all the factors in the C&E diagram can provide insight into the long-term consistent performance, reproducibility, and transferability of an assay. If the problems are too severe, the choice may be made to not further evaluate an assay as it is currently described.

### Assay design

To improve confidence in the assay result, it is possible to incorporate one-time preliminary control experiments, periodic control measurements (e.g., daily, weekly, or monthly), and in-process control measurements (performed each time an assay is performed) into a method. Testing control measurements is important prior to using a new lot or batch of solvent, reagent, or consumable that could affect the results. If it is not possible to perform all the in-process control measurements concurrently, such as for an inhalation assay with a limited number of sample ports, periodic control measurements may be necessary; these are performed at a predetermined frequency but not at the same time as the assay. One-time preliminary measurements can be used to assess if there may be, for example, interferences that could limit the applicability of the assay for a certain substance. One relevant example of this is carbon nanotubes absorbing the MTS assay reagents in the absence of cells, thereby causing false positive cytotoxicity results (Worle-Knirsch et al., 2006). Another example of a one-time preliminary measurement is for ELISA when evaluating particles to assess if cytokines produced by the cell can be adsorbed by the particles, thereby reducing the apparent cytokine concentration in the medium (Petersen et al., 2020). A third example of a one-time preliminary measurement is to perform a 0-h experiment immediately after the test substance is added, after which the remaining steps in the assay are performed and the results are compared to those of the negative control (no test substance added) (Petersen et al., 2022b). A fourth example is to perform preliminary experiments to assess the potential for losses in the exposure concentration during the course of an experiment such as for compounds that bind to the test plates or that are volatile.

In-process control measurements are done each time the assay is performed and typically cover sources of variability related to both the biological test article or test substance and the exposure system or approach (e.g., pipetting into the overlying media or deposition of a substance after aerosolization). The following considerations, for example, are relevant for assays performed manually using microplates. When pipetting is performed manually, it is important to consider the type of pipette used and also the pipetting direction when using a multi-channel pipette (Elliott et al., 2017). By specifying the direction, it is possible to monitor if there is a change between the earlier and later pipetting steps (Elliott et al., 2017). When using a multi-channel pipette, one can evaluate the variability within a single pipette ejection and among ejections (Elliott et al., 2017).

Another important in-process control measurement is to evaluate the potential for substances to have a fluorescent or absorbance signal similar to that of the probe molecule (Elliott et al., 2017; Guadagnini et al., 2015; Ong et al., 2014). Compounds can directly interfere with a fluorescent assay by two major mechanisms: autofluorescence, where chemicals emit light that overlaps the fluorophore spectrum, and quenching, where chemicals absorb light directly. Compounds can also interfere with luciferase-based systems by directly inhibiting luciferase enzymatic activity, or potentially via oxidation of the luciferin substrate. While some NAMs include a separation step to remove the test substance prior to analysis, it may be possible in some assays to correct for this potential interference by, for example, subtracting the signal from the test substance. Computational models have been developed to predict whether test compounds will interfere with commonly used fluorescence and luciferase-based test systems and can be applied prior to test substance selection (Borrel et al., 2020a,b).

One frequently used in-process control is the positive chemical control (Petersen et al., 2021a). This in-process control material can be used to evaluate the sensitivity of the assay response and also the maximal response (e.g., if 100% cell death or saturation of receptor binding is reached) (Nelson et al., 2013). Ten key characteristics should be considered when selecting the positive control material(s) to maximize the assay’s long-term usage, safety, and reliability: 1) the biological mechanism of action, 2) ease of preparation, 3) chemical purity, 4) verifiable physical properties, 5) stability, 6) ability to generate responses spanning the dynamic range of the assay, 7) technical or biological interference, 8) commercial availability, 9) user toxicity (i.e., potential toxicity to operators), and 10) disposability (Petersen et al., 2021a). For some assays, using multiple positive control materials that have different responses (e.g., weak or strong) in the assay may be desirable. Other assays may use a single positive control material and test multiple concentrations (Elliott et al., 2017). If only a single concentration of a single positive control material is used, this will not be sufficient to fully model the assay positive control concentration-response with a Hill model, which contains at least three parameters, because only two data points will be available (i.e., negative control and positive control material dose).

One key decision in the assay design is the number of replicates for the test substances and the different in-process control measurements. In-process controls are often performed in a multiwell plate with the test sample. This choice can be informed by data gathered during the within-laboratory evaluation and statistical analysis to assess the performance improvement (e.g., decrease in standard deviation values) for each additional replicate. In general, testing larger numbers of in-process control measurements and replicates for each will reduce the number of test substances that can be evaluated and may increase the cost to perform the assay. There may also be tradeoffs in terms of what in-process control measurements to include in the assay protocol as it may not be possible to evaluate all potential in-process control measurements. If a step in the protocol or a source of variability is not evaluated, there is no evidence that this aspect of the assay is performing as expected. Therefore, careful consideration, ideally via C&E analysis, should be applied to determine the expected key sources of variability to monitor. Repeating the experiment on multiple days is necessary to understand the variability over time. Both the number of repetitions and the number of replicates can depend upon the target variability for the result. To have greater statistical confidence, higher numbers of repetitions and replicates may be needed.

### Check sheets: track supply chain and protocol metadata

Check sheets can be used to record key metadata. This is important for good laboratory practice (GLP) (WHO, 2001); understanding why the assay may not be performing as expected (e.g., based on the results for the in-process control measurements); making the metadata available using findable, accessible, interoperable, and reusable (FAIR) guidelines (Wilkinson et al., 2016); and to support troubleshooting. For example, if one of the in-process control measurements differed from the historical range, reviewing the assay check sheets could reveal if a different lot number was used for a key reagent. This could then be evaluated using additional experiments to compare results for this in-process control measurement using different lot numbers for this reagent.

For some assays, data calculators can be designed that request certain pieces of metadata, extract the data for each in-process control measurement, evaluate if the run successfully achieved all specifications, and potentially perform statistical calculations to evaluate the results for the test substances. An example of an advanced check sheet from the electrophilic allergen screening assay (EASA) for recording the needed metadata and verifying that the assay is performing according to the protocol is the data calculator provided by Petersen et al. (2022c). These check sheets can also help confirm that the assay is being performed under GLP if needed.

New tools that can help fulfill the purposes of manual check sheets, such as electronic notebooks, are becoming more widely available. These notebooks can help record relevant metadata (e.g., date, operator, information about each reagent used, experimental protocol). In addition, laboratory information management systems (LIMS) can also be used to record metadata for experiments and store the information in a centralized location. Additional key advantages of experimental electronic records over paper check sheets are that the data is searchable, easily transferred among laboratories, and that the metadata can readily be linked to specific experimental results.

## 3 Within-laboratory evaluation of assay performance

The within-laboratory evaluation utilizes the findings from the conceptual analysis step to direct measurements of different sources of variability in the assay. The overarching goal is a qualified assay with intermediate specifications for in-process control measurements and for one-time preliminary experiments. These specifications may change based on revisions to the assay and interlaboratory comparison data. In addition, the results from this stage may cause the flow charts, C&E diagrams, assay design, and check sheets to be modified.

### Control charting: detect in-process control measurement trends

**Fig. 4:**
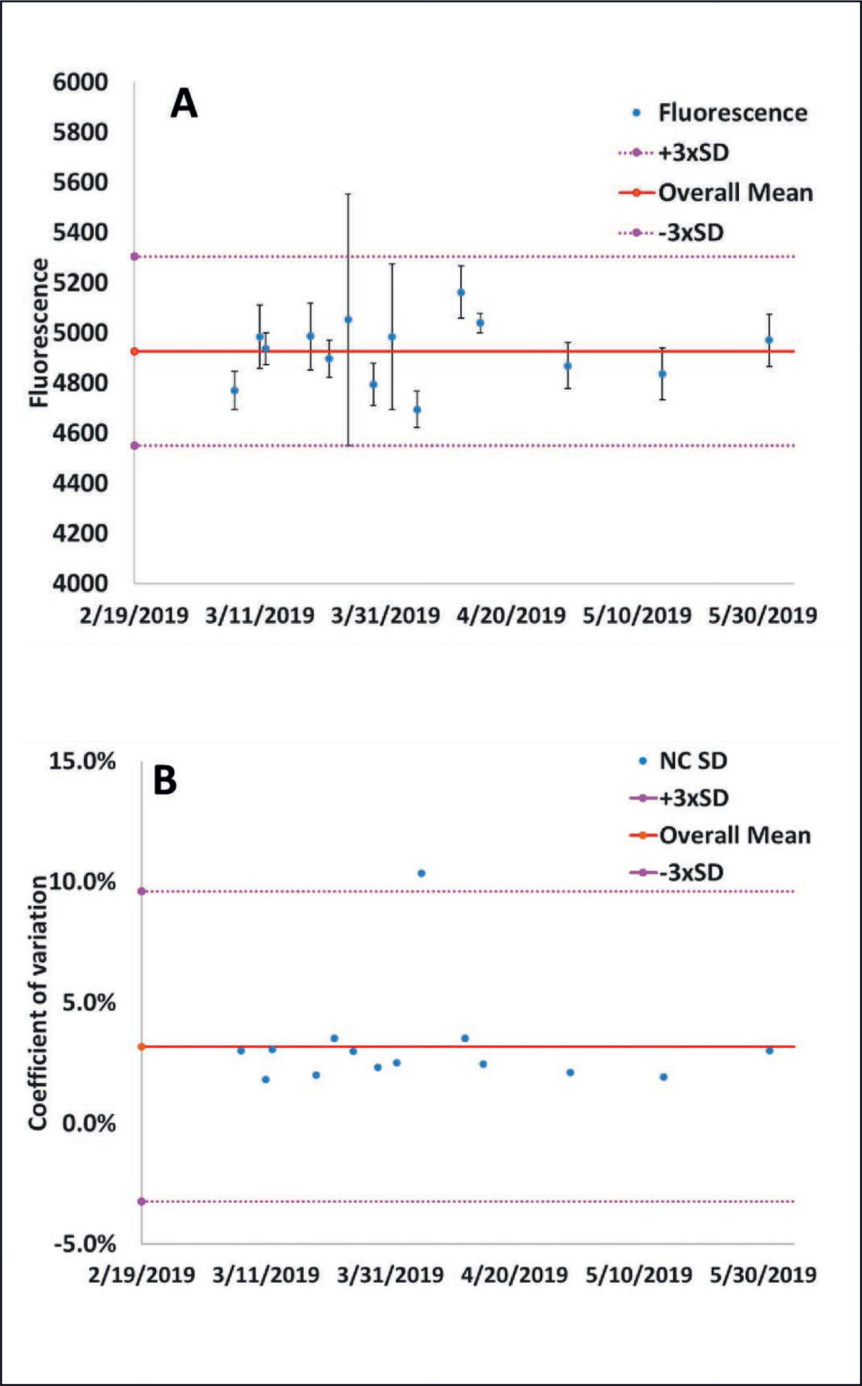
Control charting data for EASA fluorescence method for the negative incubator control (A) Mean and (B) coefficient of variation for all experiments depending on the date they were performed. Note that one value is an outlier for the coefficient of variation and outside of the specifications for this study (overall mean ± 3 times the average standard deviation value). Also, there is no systematic trend with either the mean or coefficient of variation values across time. This figure has been modified and reprinted with permission from Petersen et al. (2022c).

**Fig. 5:**
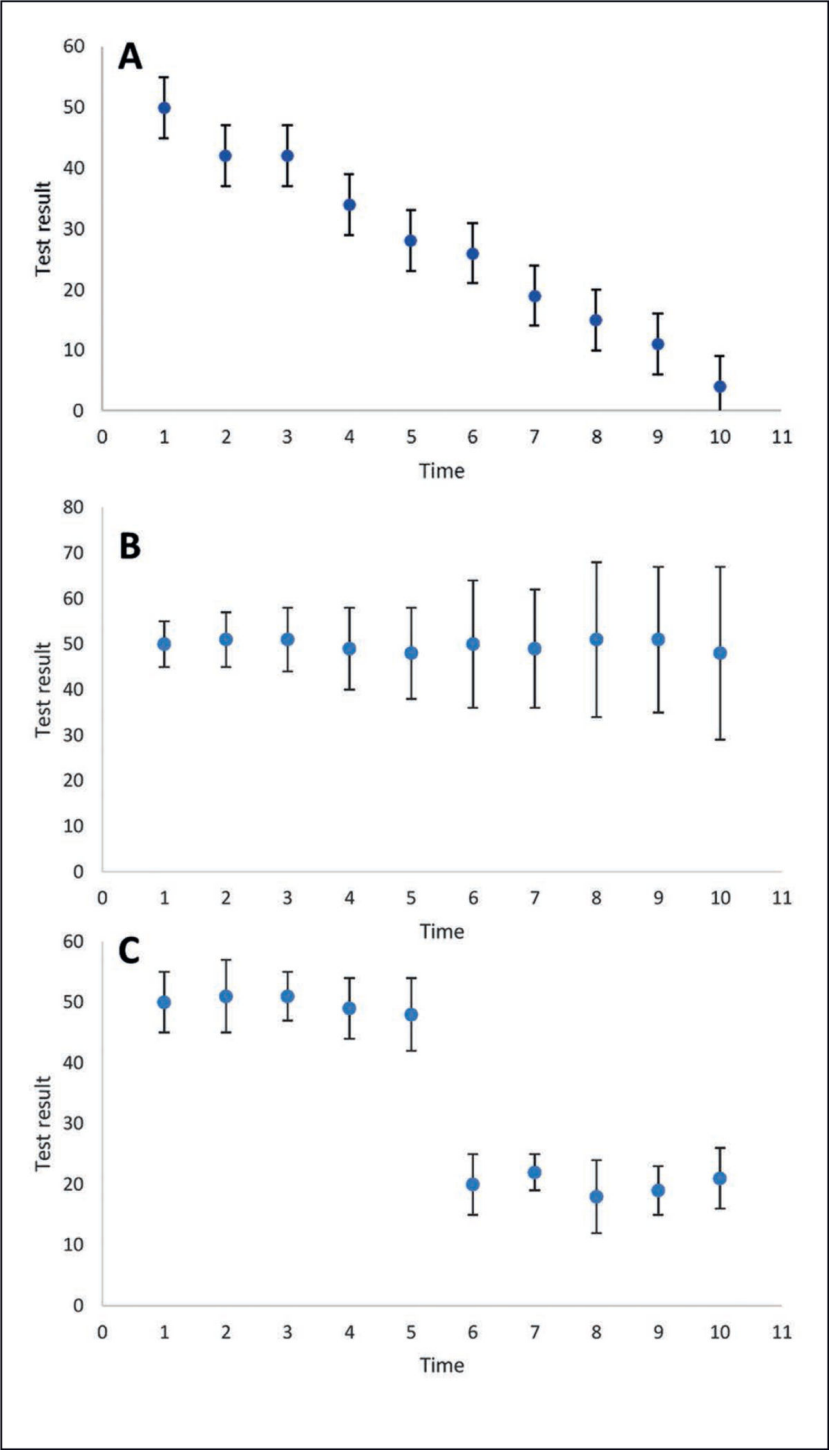
Illustrative examples of control charting data generated to show systematic trends (A) There is a decrease in the mean test result value (indicated by the blue circle) across time, (B) an increase in the uncertainty (indicated by the error bars) across time, or (C) an abrupt change in the mean value (occurring between the data points for time points 5 and 6).

Control charts should be used to monitor the mean values and variability (e.g., standard deviation values) of all in-process control measurements across time (Fig. 4, 5) to assess if there are systematic changes across time, or if some data points (e.g., as shown in Fig. 4B) do not follow the typical trend and may be suspect (Leibrock et al., 2020; Hanna et al., 2016). If systematic changes are observed across time, as shown in Figure 5A and B, this may suggest instability in the assay, e.g., due to a degrading reagent. If abrupt changes are observed, as shown in Figure 5C, it could be helpful to use the check sheets to evaluate if there was a change in the reagents and the functioning of the pipettes. It is important to note that in-process control measurement results may not be fully independent. For example, a pipetting malfunction could impact the results for all in-process control measurements for which this pipette was used.

### Evaluate the applicability domain

Given the wide range of substances that could potentially be tested in a NAM, it may be important to test different types of substances such as dissolved chemicals, particles (e.g., engineered nanomaterials or plastic microparticles), and creams. For example, different types of substances may be topically applied at an air-liquid interface such as a cream to a 3-D skin construct, while creams are often not amenable to testing in NAMs using submerged culture. Separate testing may be desired for different classes of compounds (e.g., agrochemicals, personal care products, etc.) to compare the NAM result with *in vivo* data. A comprehensive treatment of this topic is beyond the scope of this manuscript, and readers are referred to GIVIMP (OECD, 2018, Chapter 6) for further details.

### Robustness testing

Robustness testing can be guided by the C&E diagrams created during the conceptual evaluation stage (Hanna et al., 2016) so that ideally each branch and subbranch is evaluated. This can include intentionally varying aspects of the assay such as the duration of steps, concentrations of key reagents, and testing reagents or instruments from different manufacturers. When an assay may require modifications such as changing the test medium when testing certain substances (e.g., particles), it may be necessary to evaluate the extent to which these modifications impact the results for the positive control material (Hanna et al., 2016). If changes in the positive control material results are not observed, results obtained using this modification could be comparable to data from the historical procedure. Testing the same compounds on different days can also provide insight into the reproducibility of the assay across time.

### Scatterplots: investigate interactions between in-process control measurement and test substance values

**Fig. 6:**
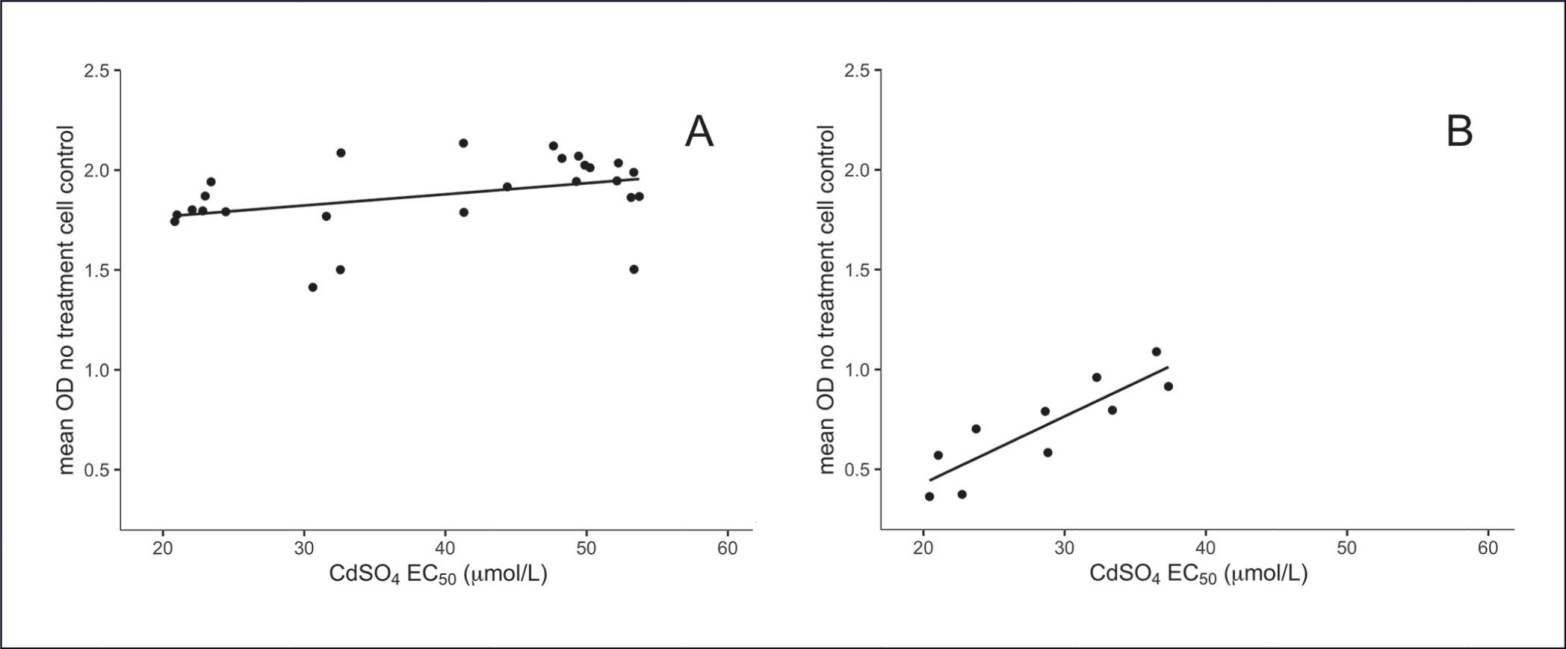
Correlation of CdSO4 EC50 values determined using the MTS assay with mean optical density (OD) negative control values using A549 cell lines These data either show a lack of an interaction (A) or an interaction (B) between the EC_50_ values depending upon the range of mean OD values. The solid lines are linear regression fits. The slope in B is statistically different from 0, indicating that the EC_50_ value is correlated with the OD values. Modified and reprinted with permission from Elliott et al. (2017).

Scatterplots are a key quality tool to evaluate whether there is a relationship among different in-process control measurements or between in-process control measurements and the results for test substances. If a relationship is observed, there is an interaction between these measurements. Figure 6 shows an example of an interaction between the EC_50_ values for the positive chemical control (CdSO_4_) and the mean optical density values, which relate to the cell number, for the negative control cells using the MTS assay (Elliott et al., 2017). To ensure reliable results, the results produced for a test substance should be independent from the results observed for the in-process control measurements as long as they remain within an acceptable range set by specifications. In the MTS assay example shown in Figure 6, the results for the negative control cells were only independent from the positive chemical control test results when outlier results, caused by differences in performing washing steps, were excluded (Elliott et al., 2017). Therefore, a specification was added to the protocol to exclude results for runs with too few cells. It may be important for some assays to evaluate interactions among in-process control measurements using a comprehensive design of experiment approach whereby different factors are intentionally varied in different combinations. This could potentially be performed using automation to test a broader range of factors than would be feasible manually.

### Histograms: visualize data distribution characteristics

**Fig. 7:**
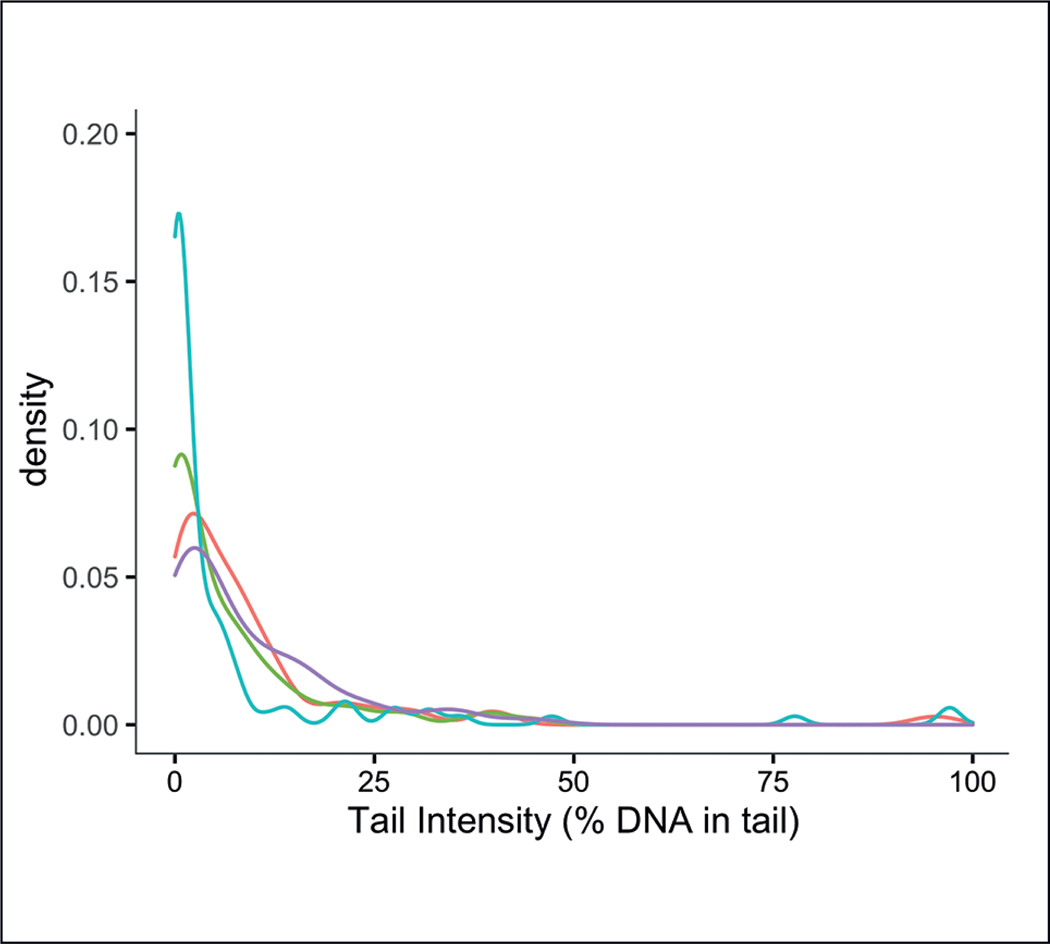
A549 cells were exposed to a 10 mmol/L dose of ethyl methanesulfonate (EMS) for 30 minutes Four independent experiments with two technical replicates each were performed. Results from the same experiments are shown in the same color as kernel density plots. The figure and figure caption are modified and reprinted with permission from Cassano et al. (2020).

Plotting the results from in-process control measurements using histograms, or other approaches such as kernel density distributions, can provide insight into the distribution of the obtained results. The assumption that the data from in-process control measurements follow a Gaussian distribution is often made when designing statistical models or performing statistical analyses, but it is important to check this assumption in case the distribution is skewed or non-Gaussian (e.g., by plotting the data or using the Shapiro-Wilk test). For example, data from the Comet assay obtained from cells exposed to different doses of the positive control ethyl methanesulfonate (EMS) were clearly non-Gaussian for the lowest exposure concentrations (Fig. 7) (Cassano et al., 2020). For non-Gaussian data, different statistical distributions, or nonparametric analyses, may be needed. The information from histograms can also be helpful in setting specifications. For example, histograms of data from the “bubble” control for the electrophilic allergen screening assay (EASA), an *in chemico* skin sensitization assay, revealed the typical distribution of data for this control measurement and supported setting a threshold to eliminate outlier data, thereby limiting the amount of potential bias in the assay results from bubbles (Petersen et al., 2022c).

## 4 Statistical data analysis

Data from the within-laboratory testing enables the use of statistical models to quantify and compare assay results. For example, statistical approaches that use replicate control and test substance measurements can yield probabilistic information for decision-making such as the likelihood of a false positive or false negative call. Based on the statistical analysis of the results from the within-laboratory evaluation, it may be necessary to reevaluate the assay design and the C&E diagram.

**Fig. 8:**
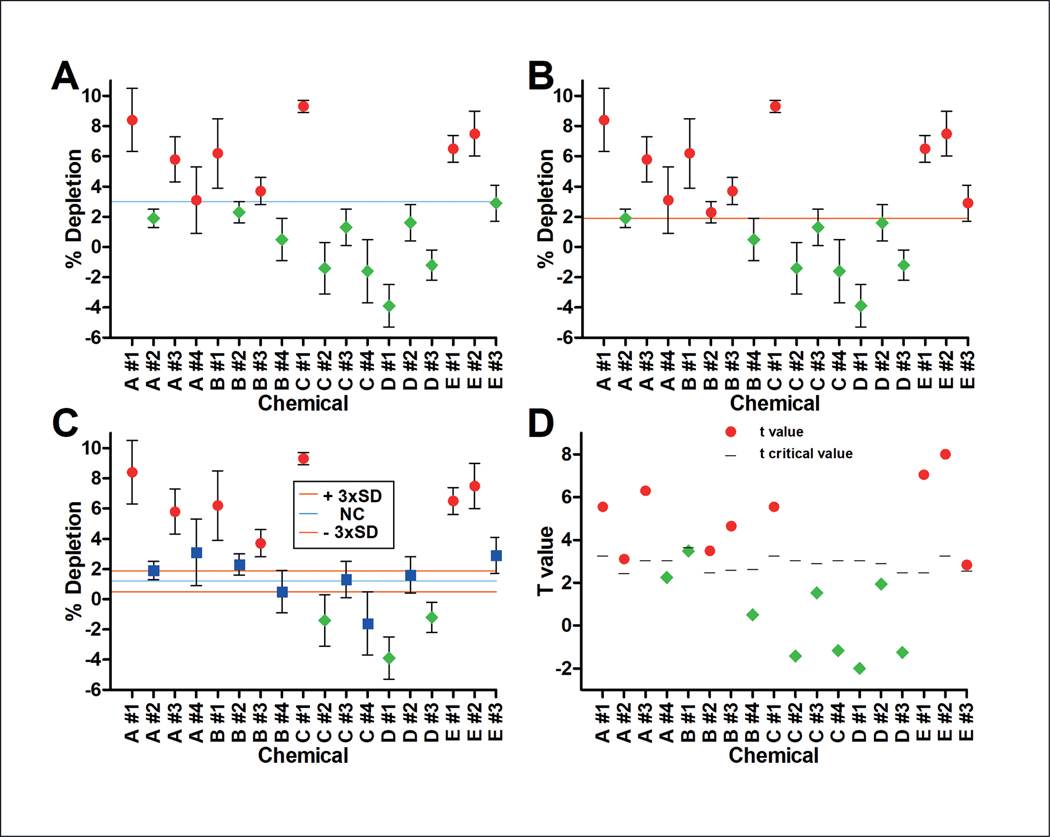
Data showing different approaches for evaluating test data Data from the EASA method that was close to the positive/negative threshold was used. The approaches for evaluating the same data from the EASA method were (A) a static call line of 3%, (B) and (C) a call line of three times the standard deviation of the negative control, and (D) a variable call line based on the critical t value for that particular compound and run. For the data in parts A, B, and C, the data symbols represent the mean values, and the error bars are the 95% confidence interval of the uncertainty for the test chemical data only. For the data in (D), the data represent the t values. Data in red circles, green diamonds, or yellow squares are “positive,” “negative,” or “borderline”, respectively. For (A), data are “positive” or “negative” if the value is above or below the static call line or 3%, respectively. For (B), data are “positive” or “negative” based on if the data point is above or below a call line of the mean negative control value for all runs plus three times the standard deviation of the mean value for those runs. For (C), data are “positive” or “negative” based on if the data point and its error bars are fully above or below the mean negative control value plus or minus three times the standard deviation, respectively. Values for which the error bars are within this range are considered “borderline”. For (D), values are “positive” or “negative” if the t values are above or below the t critical value. The data used in this figure was published in Petersen et al. (2022c) and is reprinted with permission.

Figure 8 shows selected data from the EASA method (Petersen et al., 2022c) for compounds near the positive/negative threshold that were evaluated with multiple statistical approaches. Different criteria for assessing “positive” or “negative” results can greatly affect how a test result is interpreted. If the mean values for the test results were compared with a fixed value of 3% (Fig. 8A), mean values that are slightly above this call line are positive (e.g., Chemical A #4) while mean values slightly below this call line are negative (e.g., Chemical E #3). If threshold values are based on variability in the negative control (e.g., 3 times the standard deviation), different calls can result (Fig. 8B).

An alternative approach (Fig. 8C) is to label values as “borderline” if the 95% confidence intervals for the test compound overlap with the threshold value. This approach considers uncertainty in the measurement value not accounted for in Figure 8A and B. Many of the data points shown are labeled “borderline” using this approach. A recently published OECD Guideline on Defined Approaches for Skin Sensitization (DASS) includes such an assessment of borderline results in the NAM information sources that comprise the DASS (OECD, 2021).

Statistical approaches that include all the major sources of uncertainty that could affect the outcome of the result can provide a high-quality quantitative description of a measurement result for optimal decision calls. For this example with this EASA method, that would include the uncertainty from the wells for the negative control, test compound, solvent control, and test compound blank (Petersen et al., 2022c). This aggregate uncertainty can then be used to calculate tcritical values for a certain value of α using a frequentist approach (Fig. 8D). An additional advantage of this approach compared to that in Figure 8C is that it captures the uncertainty specific to this compound in a particular run, while the approach in Figure 8C uses average values for the negative control wells across all runs, which would not reflect the uncertainty in an individual assay.

## 5 Determination of method transferability

For some contexts of use, such as if the transferability of the assay is required, it may be necessary to perform interlaboratory testing. Interlaboratory testing can yield numerous advantages in improving a NAM such as the following: evaluating the transferability of the NAM; identifying steps of the protocol that could be misinterpreted; developing more robust specifications for in-process control measurements as compared to those set by within-laboratory testing; and comparing the within- versus between-laboratory variability to reveal the extent to which the assay is harmonized (i.e., within-laboratory variability is similar to the total variability). All of this information can be used to refine and improve the protocol and further develop standard operating protocols (SOPs), assay guidelines, and training modules. However, there are many methods that are not amenable to being transferred between laboratories (e.g., due to proprietary materials, need for specialized instrumentation or training). In such cases, the robustness and reliability of these NAMs may still be satisfactorily demonstrated via the technical framework described here.

## 6 Conclusions

The use of this technical framework, and the tools highlighted herein, can support the development of robust NAMs that can meet a wide variety of research and regulatory needs. The metadata collected through this process, such as through the check sheets and in-process control measurements for each experiment, also support the application of FAIR principles and broader usage of the data collected. In addition, having a thorough understanding of the expected values and their variabilities for the in-process control measurements supports troubleshooting the assay when problems arise during its long-term usage. The modeling considerations will generate a statistical understanding of the assay results that can yield probabilistic information for different decision choices. These steps can facilitate developing the requisite documentation to conduct NAMs using GLP, or in the spirit of GLP, and ultimately establish harmonized test guidelines for widespread use.

## Figures and Tables

**Tab. 1:** Selected examples of artifacts and biases revealed by quality control measurements in NAMs

Issue detected	Role of quality control measurements
A549 cells obtained from two companies yielded different toxicity results.	Short tandem repeat measurements revealed genetic differences (Elliott et al., 2017).
Negative control values abruptly showed different estrogenicity values.	Check sheets revealed that a new lot of cell culture flasks was the cause of the difference; discussions with the manufacturer revealed that there were changes to the flask formulation despite the product number being unchanged (unpublished data).
Negative control values for a probe molecule decreased with each subsequent pipetting step.	Comparing negative control values among sequential pipetting steps and robustness testing helped reveal that photodegradation had occurred (Petersen et al., 2022c).
One laboratory in an interlaboratory comparison yielded substantially different toxicity values.	In the outlier results, values for the negative control wells had fewer cells compared to the other laboratories, which led to lower EC_50_ values in wells with fewer cells (Elliott et al., 2017).
Photoactive nanoparticles biased Comet assay results.	In studies with TiO_2_ nanoparticles, control experiments testing the gel electrophoresis step (after cellular exposure) in either the laboratory light or dark revealed differing results, potentially as a result of laboratory-light induced photoactivation (Petersen et al., 2014; Gerloff et al., 2009).

## Data Availability

No datasets were generated or analyzed in this study.
